# KLF4 Initiates Dedifferentiation of Systemic Sclerosis Lung Fibroblasts

**DOI:** 10.3390/cells15100921

**Published:** 2026-05-18

**Authors:** Ludivine Renaud, Samantha Kotz, Aravind Menon, Carol Feghali-Bostwick

**Affiliations:** 1 Department of Medicine, Division of Rheumatology and Immunology, Medical University of South Carolina, 96 Jonathan Lucas Street, Suite 822, Charleston, SC 29425, USA; renaudl@musc.edu (L.R.); samantha.kotz@tufts.edu (S.K.); 2 Department of Medicine, Division of Pulmonary, Critical Care, Allergy, and Sleep Medicine, Medical University of South Carolina, 96 Jonathan Lucas Street, Suite 816, Charleston, SC 29425, USA; menona@musc.edu

**Keywords:** scleroderma, systemic sclerosis, fibroblast markers, differentiation, dedifferentiation, RNA sequencing, fibrosis, inflammation

## Abstract

Background: Systemic sclerosis (SSc) is a systemic autoimmune disease leading to extensive fibrosis of the skin and many visceral organs, including the lungs. The need for effective treatments is urgent, as none exists today that can stop or reverse the progression of fibrosis. We examined the effect of restoring KLF4 levels in primary human lung fibroblasts of SSc patients with pulmonary fibrosis as a potential therapeutic strategy. Methods: We restored KLF4 levels in SSc lung fibroblasts by adenoviral infection and extracted total RNA for RNA sequencing. Genes and systems level analyses were performed, and selected genes of interest and markers of alveolar, inflammatory, and fibrotic fibroblasts were further validated at the mRNA and protein levels. Results: Our results showed that restoring KLF4 levels in SSc lung fibroblasts initiated dedifferentiation of αSMA and CTHRC1, expressing myofibroblasts by repressing markers of inflammatory and fibrotic fibroblasts while boosting markers of alveolar fibroblasts. Our data also revealed that restoring KLF4 levels prevented TGFβ1-induced fibrogenesis in normal lung fibroblasts, and reduced fibrosis in explanted human lungs in organ culture. Conclusions: Our results in human primary SSc lung fibroblasts showed that restoring KLF4 levels initiated dedifferentiation of fibrotic and inflammatory fibroblasts towards the phenotype of alveolar fibroblasts, their lineage precursors, highlighting the potential of KLF4 as a therapy to stop and reverse fibrosis.

## 1. Introduction

Systemic sclerosis (SSc), or scleroderma, is a systemic autoimmune disease that leads to extensive fibrosis of the skin and nearly all visceral organs in the body [1]. In the past three decades, SSc-associated interstitial lung disease (SSc-ILD) has been the leading cause of death amongst SSc patients due to the lack of effective disease-modifying treatments [2,3]. Because fibroblasts are the main effector cells in fibrosis [4], cell-based therapies specifically targeting fibroblasts may hold the key to more effective therapeutic strategies.

The transcription factor Krüppel-like factor 4 (KLF4) has been identified as a major anti-fibrotic factor that is downregulated in fibroblasts from fibrotic lungs and skins [5,6]. We have shown that loss of KLF4 promotes dermal fibrosis, while overexpressing KLF4 in dermal fibroblasts can block the transforming growth factor beta 1 (TGFβ1)-induced increase in the levels of the fibrotic markers collagen type I alpha 1 (COL1A1), fibronectin (FN1), alpha smooth muscle actin (αSMA, marker of myofibroblasts), and connective tissue growth factor (CTGF) [5]. Mice with a conditional deletion of KLF4 in fibroblasts exhibited worse bleomycin-induced lung fibrosis than wild type mice, and did not undergo the spontaneous resolution of lung fibrosis usually observed in this model [7].

Overexpressing KLF4 has shown promising results in preventing the development of fibrosis. Transgenic mice overexpressing KLF4 had significantly less lung fibrosis and epithelial-mesenchymal transition (EMT) compared to wild type when challenged with bleomycin, and adenoviral expression of KLF4 attenuated TGFβ1-induced EMT in alveolar epithelial cells (AECs) [6]. Additionally, overexpression of KLF4 in MRC5 differentiated into myofibroblasts by TGFβ reduced the expression of fibrotic markers [5,7]. Together these results suggest that KLF4 is an endogenous molecular brake on fibroblast activation and fibrosis. Strategies aiming to restore KLF4 basal expression are an attractive avenue of therapeutic research. Furthermore, since reduced KLF4 levels are implicated in both lung and skin fibrosis, this approach could have far-reaching benefits against fibrosis in different organs.

Inflammation contributes to the stepwise development of SSc pathology [8]. Several cytokines are involved in SSc-associated inflammation, including TGFβ and a myriad of interleukins. Interleukin 6 (IL6) levels are elevated in SSc skin fibroblasts and SSc whole lung tissues [9,10], contributing to the early phase of development of fibrosis in SSc patients [3,11]. The contribution of IL6 to SSc pathology warranted the development of FDA-approved therapy that slows the progression of lung fibrosis in SSc patients by blocking the IL6 receptor [12,13]. Note, however, that this treatment is mainly effective if delivered early to patients [13], as it does not stop or reverse existing fibrotic damage.

Recently, Tsukui et al. [14] identified collagen triple helix repeat containing 1 (CTHRC1) as a marker of a subpopulation of fibroblasts expressing the highest levels of collagens and mostly present in fibrotic lungs. In a follow-up study, they showed that CTHRC1^+^ fibroblasts differentiated from signal peptide CUB domain and EGF-like domain containing 2 (SCUBE2)-expressing alveolar fibroblasts post lung injury [15]. They proposed a model in which, upon injury, SCUBE2^+^ alveolar fibroblasts differentiate into serum amyloid A3 positive (SAA3^+^) inflammatory fibroblasts before transitioning to CTHRC1^+^ fibrotic fibroblasts [15].

The goal of our study is to assess the effect of restoring KLF4 levels in human primary SSc lung (SScL) fibroblasts, conduct gene and system-level analyses of the ensuing transcriptome, and track the markers of alveolar, inflammatory, and fibrotic fibroblasts.

## 2. Materials and Methods

### 2.1. SSc Patients, Normal Donors and Fibroblast Strain Information

In this study, human primary SSc lung (SScL) fibroblasts (n = 5) were used in cell culture to study the effect of adenoviral KLF4 overexpression. Fibroblasts were cultured from explanted lungs of SSc patients who underwent lung transplantation using the outgrowth method as we previously described [16]. The fibroblast strains used in this study originated from SSc patients of European or African ancestry between the age of 42 and 67 years old of both sexes (Table 1). All SSc patients had SSc-PF without pulmonary arterial hypertension (PA mean ≤ 25). 

Normal lung (NL) fibroblasts were derived from lung tissues of organ donors whose lungs were not used for transplantation (Table 1). The donors were of European or African ancestry except one who was of unknown ancestry, their age ranged from 19 to 63 years old, and both sexes were represented. All fibroblasts were used in early passage (P3-8).

### 2.2. Fibroblast Isolation and Culture

Fibroblasts were cultured using the outgrowth method. Specifically, lung tissues were minced and approximately 5 mm pieces were allowed to adhere to cell culture dishes for 45–60 min. Fibroblasts were allowed to migrate from the tissues. Fibroblasts were cultured in DMEM media (Mediatech Inc., Manassas, VA, USA) supplemented with 10% FBS (Sigma-Aldrich, Saint Louis, MO, USA) and 1X Antibiotic/Antimycotic (AB/AM, ThermoFisher Scientific, Waltham, MA, USA) Media was replaced 3 times/week. Fibroblasts were passaged when they reached 90% confluency. 

### 2.3. Fractionation of NL and SScL Fibroblasts

NL and SScL fibroblasts were seeded in 10-cm dishes with 10 mL of complete media (10% FBS, 1X AB/AM). Once cells were 80–90% confluent, the media was changed to serum-free media with 1X AB/AM for 24 h. Fibroblast fractionation was done using the subcellular protein fractionation kit for cultured cells (Pierce Biotechnology, Rockford, IL, USA, cat# 78840) according to the manufacturer protocol, and cytoplasmic, membrane, nuclear, and chromatin fractions were collected (Pierce Biotechnology, Rockford, IL, USA, cat# 78840).

### 2.4. KLF4 Overexpression (KLF4-OE) by Adenoviral Infection of SScL and NL Fibroblasts

KLF4 was overexpressed in SScL and NL fibroblasts using the adenovirus Ad-h-KLF4 from Vector Biolabs (cat# 1787, abbreviated ad-KLF4). As a control, the adenovirus Ad-CMV-Null (cat# 1300, abbreviated ad-Null) from the same company was used. SScL and NL fibroblasts were plated in 6-well plates (125,000 cells/well) in complete DMEM media (Mediatech Inc., Manassas, VA, USA) with 10% FBS (Sigma-Aldrich, Saint Louis, MO, USA) and 1X Antibiotic/Antimycotic (ThermoFisher Scientific, Waltham, MA, USA) and allowed to recover for at least 48 h. When 80% confluency was reached, media was removed, wells were washed 2 times with sterile 1X PBS. After the last wash, 500 µL of 1X sterile PBS was added to each well, and 1 µL of ad-KLF4 or ad-Null was added to the appropriate well(s) at a final multiplicity of infection (MOI) of 33. The plates were incubated at room temperature for 1 h and rocked gently every 15 min. After 1 h, 1 mL of complete media was added to each well and the plates were incubated at 37 °C and 5% CO_2_ for 24 h prior to changing the media.

**Figure 1:**
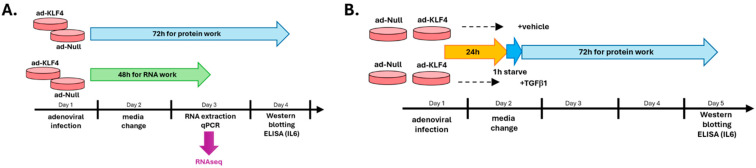
Experimental design. (**A**) Experimental design for the overexpression of KLF4 in SScL fibroblasts. (**B**) Experimental design for the overexpression of KLF4 in NL fibroblasts followed by TGFβ1 stimulation. NL fibroblasts were infected for 24 h with ad-KLF4 or ad-Null in complete media, then the media was changed to serum-free media for 1 h prior to TGFβ1 stimulation for 72 h.

For SScL fibroblasts (Figure 1A), 24 h post adenoviral infection, the media was changed with complete media. 48 h post adenoviral infection, media was removed, and each well was washed with cold 1X PBS. RNA was extracted using 500 µL of Trizol (Invitrogen, Carlsbad, CA, USA) following the manufacturer’s instructions. For protein extraction, fibroblasts were culture for 72 h post adenoviral infection. Conditioned media was saved, and each well was washed with cold 1X PBS. Lysates were harvested by scraping in 200 µL of 2X SSB (125 mM Tris pH 6.8, 10% glycerol, 3% SDS, 0.01% bromophenol blue, 715 mM beta-mercaptoethanol). The media were centrifuged at 5000 rpm for 5 min to remove debris and 100 µL was transferred to a new tube with 20 µL of 6X SSB. All samples in SSB were boiled at 105 °C for 10 min and stored at −80 °C. 

For NL fibroblasts (Figure 1B), 24 h post adenoviral infection, the complete media was removed, the wells were rinsed 3 times with serum-free media (no FBS, 1X AB/AM) and incubated with serum-free media for 1 h. The media was then replaced with serum-free media containing 10 ng/mL TGFβ1 (R&D, Minneapolis, MN, USA, cat# 240-B-010) or vehicle BSA/HCl (R&D, cat# RB04) for 72 h. 

### 2.5. KLF4-OE by Adenoviral Infection of Human Lung Tissues in Organ Culture

Explanted human fibrotic lungs from patients with familial pulmonary fibrosis (FPF), non-specific interstitial pneumonia (NSIP) and ANCA+ vasculitis associated interstitial lung disease (AV-ILD) were processed into 4–5 mm^2^ cores and placed in 6-well plate (3 cores per well) in 1.5 mL of DMEM media with 1X Antibiotic/Antimycotic. Cores were infected with ad-KLF4 or ad-Null at MOI of 33 for 1 h at room temperature under sterile conditions with gentle rocking every 15 min (Vector Biolabs, see details above). After 1 h, the plate was moved to the incubator (5% CO_2_, 37 °C). Cores were collected after 24 h, 48 h, 72 h and 96 h and flash frozen for RNA and protein extraction. Cores were also fixed in formalin and paraffin-embedded for tissue sectioning. Cores were homogenized in 1 mL Trizol for RNA extraction or in 1 mL RIPA buffer with proteinase and phosphatase inhibitors for protein extraction using the Bead Ruptor 24 (Omni International, Kennesaw, GA, USA). 

### 2.6. cDNA and qPCR

RNA concentration and quality were assessed using a NanoDrop Lite spectrophotometer (ThermoFisher Scientific, Waltham, MA, USA). cDNA was synthesized using random hexamers and the SuperScript IV reverse transcriptase (Invitrogen, Carlsbad, CA, USA). 1 μg of RNA per 20 μL was used for cDNA synthesis on a C1000 Touch Thermal Cycler (Bio-Rad, Hercules, CA, USA).

Gene mRNA expression levels were measured by quantitative PCR (qPCR) using the TaqMan real-time PCR system (Life Technologies, Carlsbad, CA, USA) according to the manufacturer’s protocol on a TaqMan Gene Expression Assays Step One Plus real-time PCR system (Life Technologies) as previously described [17] using the following TaqMan human primers for our target genes: *KLF4* (Hs00358836_m1), *CTHRC1* (Hs00298917_m1), *IL6* (Hs00985639_m1), *MMP1* (Hs00899658_m1), *COL1A1* (Hs00164004_m1), *ACTA2* (Hs00426835_g1), *SCUBE2* (Hs00221277_m1), *GPX3* (Hs01078668_m1), *CCL2* (Hs00234140_m1), *SERPINE1* (Hs01126607_g1). The housekeeping genes *GAPDH* (FAM Hs02758991_g1, abbreviated GAP) and *B2M* (VIC Hs00187842_m1) were duplexed. 

The target gene 2^−ΔCT^ values normalized to *GAPDH* or *B2M* were statistically analyzed in GraphPad Prism version 10.4.1 (GraphPad Software Inc., La Jolla, CA, USA). 

### 2.7. RNA Sequencing and Differential Expression Analysis (DEA)

To identify genes regulated by KLF4 in human lung fibroblasts, we expressed KLF4 in SScL fibroblasts for 48 h and extracted total RNA for RNA sequencing (RNAseq). RNAseq analysis was done at Novogene Corporation Inc. (Sacramento, CA, USA) as described in details in Nguyen et al. [18]. Paired-end clean reads were aligned to the Homo sapiens grch38 reference genome using the hierarchical indexing for spliced alignment of transcripts 2 (HISAT2, version 2.2.1. The RNAseq data will be publicly available on NCBI GEO under accession # GSE317058 after 31 January 2027.

The DEA “ad-KLF4 vs. ad-Null” was done using DESeq2 [19]. DESeq2 analysis returned the false discovery rate (FDR) adjusted *p*-value (q-value) and the log2 fold change (log2FC) for each gene. Differentially expressed genes (DEGs) were identified based on two criteria of significance: q-value < 0.1 and log2FC > |0.6|. Heatmap was generated by DESeq2 from the normalized counts of all genes with a q-value below 0.1. To generate a heatmap with selected genes of interest, MORPHEUS was used (https://software.broadinstitute.org/morpheus, accessed on 18 February 2026).

### 2.8. Functional Enrichment and Pathway Impact Analyses

The DESeq2 output results were entered in iPathwayGuide (Advaita, Ann Arbor, MI, USA) and analyzed using the same criteria of significance q-value < 0.1 and log2FC > |0.6|. iPathwayGuide provides a proprietary “Impact Analysis” method that is based on “Over Representation Analysis” and “Perturbation Analysis”; for more details, see Ahsan et al. [20] and Nguyen et al. [18].

### 2.9. Western Blotting and ELISA

The conditioned media and cell lysates of the ad-KLF4 infected SScL fibroblasts were analyzed by Western blotting. Equal amounts of protein were separated by electrophoresis using 10% SDS-PAGE gels for cell lysates and 8% SDS-PAGE gels for conditioned media in 1X SDS running buffer (25 mM Tris, 250 mM Glycine and 0.1% SDS) at 100 Volts for 2 h and transferred to nitrocellulose membrane (Amersham, Marlborough, MA, USA) in transfer buffer (25 mM Tris, 192 mM Glycine and 20% MeOH) at 0.3 milli-Amps (mAmp) for 2 h on ice. Membranes for whole cell lysate samples were blocked in 5% milk in 1X TBST (20 mM Tris pH 7.6, 137 mM NaCl, 0.05% Tween 20) before overnight incubation with primary antibody at 4 °C. For membranes with conditioned media samples, SYPRO Ruby fluorescent stain (Invitrogen, Carlsbad, CA, USA, cat#S12000) was used to visualized proteins prior to blocking with milk. The following primary antibodies were used to probe for our target genes: KLF4 (R&D, Minneapolis, MN, USA, cat# AF3640), αSMA (Abcam, Waltham, MA, USA, cat# ab5694), collagen type I (Southern Biotech, Birmingham, AL, USA, cat# 1310-01), caspase-3 (Cell Signaling, Danvers, MA, USA, cat #9662), EGR1 (Cell Signaling, cat #4153) and GAPDH as the loading control (Santa Cruz, Dallas, TX, USA, cat# sc47724, abbreviated GAP). The following secondary antibodies were used: anti-goat IgG-HRP (Santa Cruz, Dallas, TX, USA, cat# sc-2354), anti-rabbit IgG-HRP (Sigma-Aldrich, St Louis, MO, USA, cat# GENA9340), anti-mouse IgG-HRP (Promega, Madison, WI, USA, cat# W4021). Blots were imaged using the enhanced chemiluminescent (ECL) horseradish peroxidase (HRP) substrate SuperSignal West Pico PLUS (ThermoFisher, cat# 34580) on the iBright 750 imaging system (ThermoFisher Scientific, Waltham, MA, USA). Levels of IL6 protein were quantified in conditioned media using ELISA (EH2IL6; Invitrogen, Carlsbad, CA, USA) at absorbance 450 nm on a SYNERGY H1 Microplate Reader (BioTek, Winooski, VT, USA).

### 2.10. MTT Proliferation Assay

To assess cell proliferation, 10,000 cells/well were plated in 100 μL complete media in a 96-well plate. Once 80–90% confluency was reached, cells were infected with the KLF4-adenovirus or null-adenovirus (n = 15) as previously described in 33 μL of PBS/well. After the 1 h incubation time, 70 μL of complete media was added to each well. The media was changed the next day, and the CytoSelect MTT Cell Proliferation Assay (CELL BIOLABS, Inc., San Diego, CA, USA, cat# CBA-252) was performed 72 h post infection according to manufacturer’s protocol. Briefly, 10 μL of CytoSelect MTT Cell Proliferation Assay Reagent was added to each well and the plate was incubated at 37 °C and 5% CO_2_ for 3 h. Then 100 μL of Detergent Solution was added to each well and incubated at room temperature in the dark for 3 h. Absorbance was read at 550 nm on a SYNERGY H1 Microplate Reader (BioTek, Winooski, VT, USA).

### 2.11. Human Single Cell RNA Sequencing Data Processing

Raw count matrices from GSE132771 and from GSE128169 were downloaded as sparse .mtx files together with corresponding gene and barcode annotations. For each cell, we computed the number of detected genes, total UMI counts, and mitochondrial transcript percentage. Cells were retained if they satisfied: nFeature_RNA > 500; nCount_RNA < 100,000; percent.mt < 12%. These thresholds removed low-quality, dying, or doublet-like cells. Filtered cells were normalized using NormalizeData, variable features were identified with FindVariableFeatures, and data were scaled using ScaleData. Principal component analysis (PCA) was performed on the scaled matrix. To correct for sample-specific effects, we applied CCA-based integration using Seurat’s IntegrateLayers with method = CCAIntegration. The integrated assay was reconstructed using JoinLayers, and the integrated PCA space was used for neighbor graph construction (FindNeighbors) and clustering (FindClusters, resolution = 0.5). UMAP embeddings were computed using the integrated reduction. Major lineages were identified using canonical markers for epithelial (EPCAM, KRT8/18), endothelial (PECAM1, VWF), immune (PTPRC, LYZ, CD3D), and fibroblast (COL1A1, DCN, LUM, PDGFRA) populations. Module scores for each lineage were computed using AddModuleScore. Fibroblasts were isolated by selecting cells with high fibroblast scores (FB_POS1 > 0.5) and low epithelial, immune, and endothelial scores (EPI1, IMM1, ENDO1 < 0.1). The fibroblast subset was re-normalized, re-scaled, and re-embedded using PCA, UMAP, and clustering. 

Fibroblast subtypes were assigned using marker sets defined by Tsukui et al. (2024) [15] (alveolar, inflammatory, fibrotic fibroblasts). Module scores for each subtype were computed, and each cell was assigned to the subtype with the highest score. Subtype proportions were quantified per condition (normal vs SSc-ILD). Endogenous KLF4 expression was extracted per cell and compared across the six subtype × condition groups. Summary statistics (mean, median, SEM) were computed using dplyr. Violin plots and boxplots were generated to visualize distributional differences. To quantify the contribution of each fibroblast subtype to the overall KLF4 signal, we computed a weighted expression metric integrating both expression intensity and subtype abundance. 

KLF4 overexpression (KLF4-OE) signature projection: Differentially expressed genes from KLF4 overexpression experiments (UP and DOWN sets) were combined into a single signature (KLF4_OE_ALL). Module scores were computed using AddModuleScore, generating a per-cell KLF4-OE signature score. These scores were compared across fibroblast subtypes and conditions using violin plots and summary statistics. Mean KLF4-OE signature per subtype × condition group was z-scored across groups to highlight relative enrichment patterns (group-mean z-score). This normalization enabled comparison of endogenous KLF4 patterns with the KLF4-OE transcriptional program.

### 2.12. Collagen Volume Fraction from Picro Sirius Red Stained Tissue

Tissue sections were stained using Picro Sirius Red (PSR) at the MUSC Histochemical Core Lab. The stained tissues from ad-Null or ad-KLF4 infected cores were then imaged under polarized light under dark field optics to detect birefringent collagen fibers. Several fields were imaged at random from each core (n = 8–12) and analyzed with Fiji (Fiji is just ImageJ) software version 1.54p (https://fiji.sc, accessed on 18 February 2026). The collagen volume fraction was determined based on the average of the % area. 

### 2.13. Statistical Analysis

GraphPad Prism 10 version 10.4.1 (GraphPad Software, Inc.) was used for statistical analysis. To identify outliers, the ROUT method was used. When applicable, we used a two-tailed unpaired *t*-test with 95% confidence level, a two tailed paired *t*-test with 95% confidence level, or a RM one-way ANOVA with uncorrected Fisher’s LSD comparisons. *p* < 0.05 was considered significant: * *p* < 0.05, ** *p* < 0.01, *** *p* < 0.001, **** *p* < 0.0001. Error bars = SEM.

## 3. Results

### 3.1. KLF4 Levels in Human Primary SScL and NL Fibroblasts After TGFβ1 Stimulation

**Figure 2:**
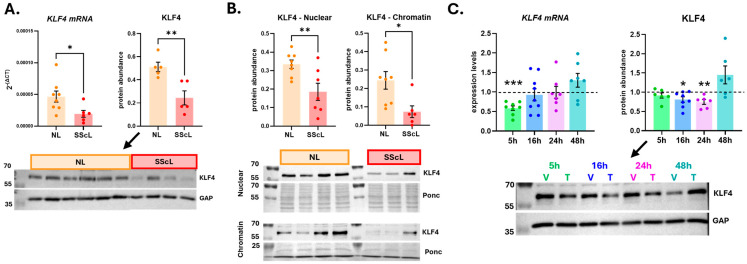
KLF4 levels in human primary SScL fibroblasts. (**A**) KLF4 mRNA (**left**) and protein (**right**) levels in NL and SScL fibroblasts. A representative Western blot is shown below the graphs. (**B**) KLF4 protein abundance in the nuclear (**left**) and chromatin (**right**) fractions of NL and SScL fibroblasts. A representative Western blot is shown below the graphs. A two-tailed unpaired *t*-test with 95% confidence level was performed on the datasets of panel (**A**,**B**), * *p* < 0.05, ** *p* < 0.01 relative to NL. (**C**) Effect of TGFβ1 stimulation on KLF4 mRNA (left) and protein (right) levels at 5 h, 16 h, 24 h and 48 h. The dashed line represents vehicle control as each dataset is relative to its own vehicle. A representative Western blot is shown below the graphs (indicated by an arrow). A two-tailed paired *t*-test with 95% confidence level was performed. * *p* < 0.05, ** *p* < 0.01, *** *p* < 0.001 relative to vehicle (dashed line). V: vehicle, T: TGFβ1.

We first quantified baseline levels of KLF4 in SScL and NL fibroblasts. Human primary SScL fibroblasts have significantly lower KLF4 mRNA and protein levels compared to normal lung (NL) fibroblasts (Figure 2A). KLF4 abundance is also lower in the nuclear and chromatin fractions of SScL fibroblasts than in NL fibroblasts (Figure 2B). In TGFβ1-stimulated NL fibroblasts, KLF4 was significantly decreased at the mRNA level after 5 h, and intracellularly at the protein level after 16 h, an effect that persisted to 24 h post TGFβ1 stimulation (Figure 2C).

### 3.2. KLF4 Overexpression in Human Primary SScL Fibroblasts Initiates Dedifferentiation

**Figure 3:**
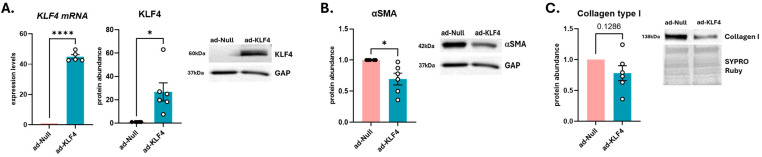
KLF4-OE in human primary SScL fibroblasts. (**A**) Efficiency of adenoviral infection of KLF4 (ad-KLF4) in SScL fibroblasts after 48 h (mRNA) and 72 h (protein, representative blot shown below). (**B**,**C**) Effect of ad-KLF4 on αSMA and collagen type I in SScL fibroblasts (representative blots shown beside). GAP was used to normalize KLF4 and αSMA in whole cell lysates, SYPRO Ruby stain was used to normalize collagen type I in conditioned media. A two-tailed paired *t*-test with 95% confidence level was performed on all datasets. * *p* < 0.05, **** *p* < 0.0001 relative to ad-Null.

Since SScL fibroblasts derived from fibrotic lungs retain the differentiated phenotype of fibrosis [3,17], we investigated whether KLF4 overexpression (KLF4-OE) in SScL fibroblasts could reverse this phenotype and initiate dedifferentiation as defined by Fortier et al. [18]. KLF4 was overexpressed in SScL fibroblasts by adenoviral vector for 48 h (RNA) and 72 h (protein) (Figure 1A). Adenoviral efficiency of KLF4 adenovirus (ad-KLF4) was confirmed at both mRNA and protein levels (Figure 3A). KLF4 overexpression significantly decreased αSMA (Figure 3B) and noticeably decreased collagen type I protein abundance in SScL fibroblasts, albeit not significantly due to one cell line not responding well (Figure 3C). Together, our results are consistent with myofibroblasts undergoing dedifferentiation.

### 3.3. KLF4-Driven Transcriptome and Functional Enrichment

**Figure 4:**
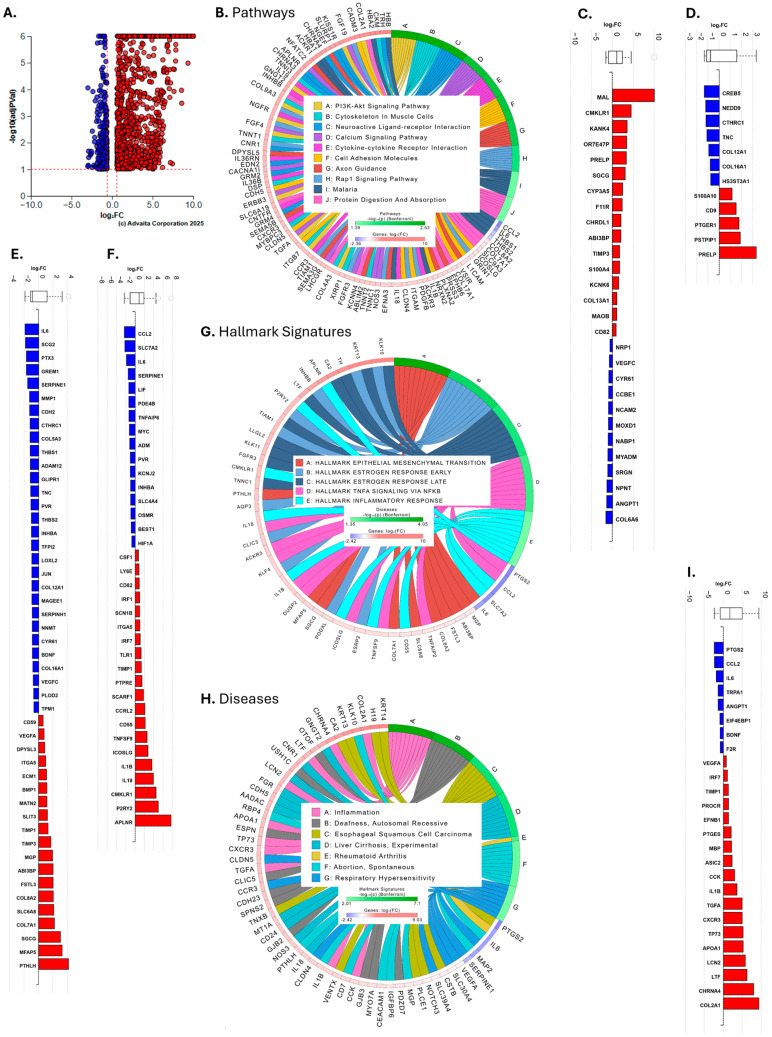
KLF4-driven pathway and functional enrichment. (**A**) Volcano plot showing all DEGs (q < 0.1, log2FC > |0.6| illustrated by red dashed line). (**B**) Ribbon plot of the top 10 most enriched PATHWAYS with the top 10 DEGs contributing to the enrichment. (**C**,**D**) Barplots showing the fold change in the DEGs part of the “Travaglini lung alveolar fibroblast” and “Travaglini lung myofibroblast” cell signatures, respectively. (**E**,**F**) Barplots showing the fold change in the DEGs part of the HALLMARKS “epithelial mesenchymal transition” and “inflammatory response,” respectively. (**G**) Ribbon plot of the top 5 most enriched HALLMARKS with the top 10 DEGs contributing to the enrichment. (**H**) Ribbon plot of the top 7 most enriched DISEASES with the top 10 DEGs contributing to the enrichment. (**I**) Barplot showing the fold change in the DEGs part of the DISEASE “Inflammation.” In the volcano plot and barplots, red means upregulation of gene expression and blue means downregulation.

To identify additional genes regulated by KLF4 in human lung fibroblasts, we expressed KLF4 in SScL fibroblasts for 48 h and extracted total RNA for RNAseq. Gene counts from RNAseq analysis were entered in DESeq2 along with the groups to run the differential expression analysis (DEA) “ad-KLF4 vs. ad-Null” in SScL fibroblasts. DESeq2 generated PCA plot and heatmap (Supplemental Figure S1) showed a good clustering of the samples according to ad-KLF4 or ad-Null. The DEA returned 1530 DEGs (q < 0.1, log2FC > |0.6|), out of which 1166 were upregulated and 364 were downregulated in ad-KLF4 infected SScL fibroblasts (Figure 4A, Supplemental Table S1). This dataset was entered in iPathwayGuide for functional enrichment and pathway impact analyses.

In summary, 74 pathways were significantly impacted (Supplemental Table S2). The top 10 most enriched pathways are shown in Figure 4B along with the top 10 DEGs contributing to the enrichment. Amongst these impacted pathways are the “PI3K-Akt signaling pathway”, the “Calcium signaling pathway”, “Cytokine-cytokine receptor interaction”, “Cell adhesion molecules”, and “Rap1 signaling pathway”.

In the Molecular Signatures Database (MSigDB), curated gene sets derived from single-cell RNA sequencing (RNAseq) studies, our dataset matched the transcriptomic signature of “Travaglini lung alveolar fibroblasts cell” and “Travaglini lung myofibroblast cell” (Figure 4C,D) [21]. In the alveolar fibroblast set (Figure 4C), several DEGs are upregulated, including PRELP, a marker of alveolar fibroblasts [15]. In the myofibroblast set (Figure 4D), several DEGs were downregulated, including CTHRC1 and TNC, both markers of fibrotic fibroblasts [15].

From the MSigDB Hallmarks category, the “hallmark epithelial mesenchymal transition” and “hallmark inflammatory response” were in the top 5 most enriched hallmarks (Figure 4E–G) along with hallmarks related to estrogen response (early and late) and “TNFA signaling via NFKB” (Figure 4G). The downregulation of IL6, GREM1, SERPINE1, CTHRC1, THBS1, TNC, THBS2, and PLOD2 contributes to the enrichment of the EMT hallmark (Figure 4E), and the downregulation of CCL2, IL6, SERPINE1, MYC, and HIF1A contributes to the enrichment of the inflammatory response hallmark (Figure 4F), as examples.

In another enrichment layer that links our DEGs to known disease associations curated from biomedical databases, the most enriched disease listed in that category was “Inflammation” (Figure 4H,I). Noticeably, the downregulation of PTGS2, CCL2, IL6, TRPA1, ANGPT1, EIF4EBP1, BDNF, and F2R participates in this enrichment (Figure 4I). Amongst the top 5 most enriched diseases were also “Rheumatoid arthritis” and “Respiratory hypersensitivity” (Figure 4H).

The upregulation of markers of alveolar fibroblasts (Figure 4C) and the downregulation of several myofibroblast, EMT, and inflammatory markers (Figure 4D–I) suggest that overexpression of KLF4 in SScL fibroblasts induced a phenotype shift from myofibroblasts towards alveolar fibroblasts consistent with dedifferentiation. Overall, this enrichment suggests that inflammatory and fibrotic markers are decreasing when KLF4 levels are restored in SScL fibroblasts.

### 3.4. KLF4-OE Shifts Fibroblasts Towards a Less Fibrotic and Inflammatory Phenotype

The landmark study conducted by Tsukui et al. [15] reported that the main progenitors to the inflammatory and fibrotic subpopulations of fibroblasts in human lungs are the alveolar fibroblasts present at the time of injury. Their pseudotime analysis emphasized the differentiation from alveolar fibroblasts into inflammatory fibroblasts towards the fibrotic type.

**Figure 5:**
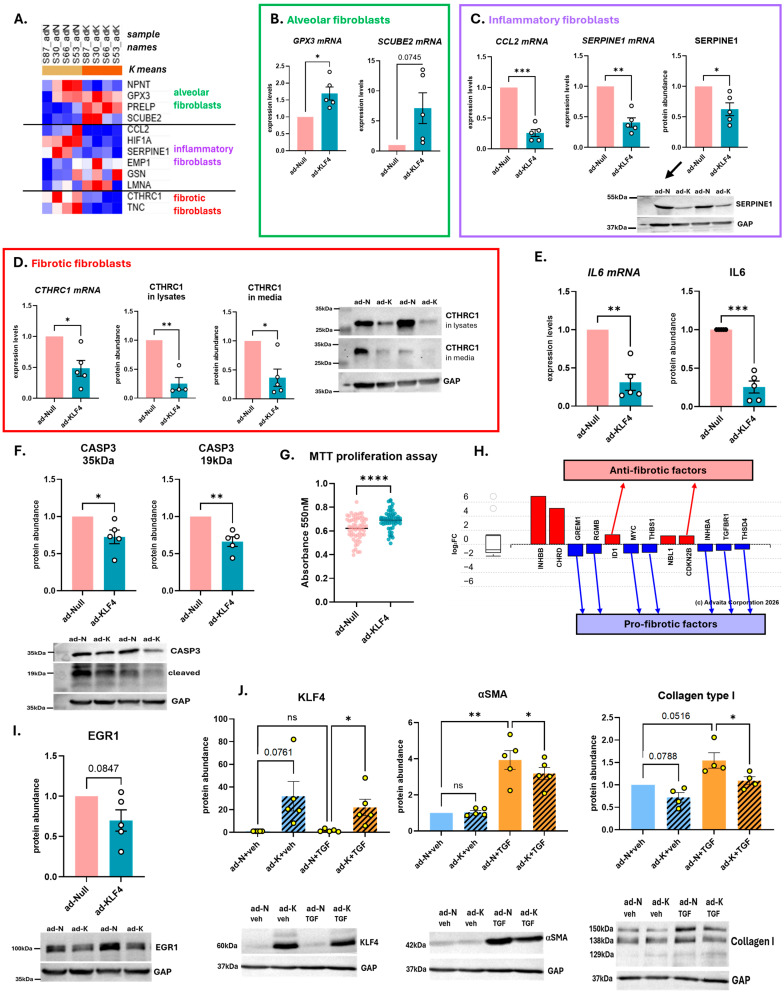
KLF4-OE shifts fibroblasts towards a less fibrotic and inflammatory phenotype. (**A**) Heatmap of alveolar, inflammatory, and fibrotic fibroblast markers based on the counts of our DEGs. Red: upregulated gene, blue: downregulated gene. (**B**) Validation of two markers of alveolar fibroblasts, *GPX3* and *SCUBE2*, by qPCR in ad-KLF4-infected SScL fibroblasts. (**C**) Validation of two markers of inflammatory fibroblasts, *CCL2* and *SERPINE1*, by qPCR in ad-KLF4-infected SScL fibroblasts. SERPINE1 was further validated in conditioned media by Western blotting (see representative blots below the graph). (**D**) Validation of CTHRC1, a marker of fibrotic fibroblasts, by qPCR and Western blotting (whole cell lysates and conditioned media) in ad-KLF4-infected SScL fibroblasts (see representative blots on the right). (**E**) Validation of IL6 by qPCR and ELISA of conditioned media of ad-KLF4-infected SScL fibroblasts. (**F**) Assessment of apoptosis via quantification of caspase 3 (CASP3) full form (35 kDa) and cleaved form (19 kDa) by Western blotting (see representative blots below). (**G**) Assessment of proliferation via MTT assay in ad-KLF4 infected SScL fibroblasts (n = 4 cell lines, 15 wells per group for ad-KLF4 and ad-Null). A two-tailed paired *t*-test with 95% confidence level was performed on the datasets. * *p* < 0.05, ** *p* < 0.01, *** *p* < 0.001, **** *p* < 0.0001 relative to ad-Null SScL fibroblasts; ns: not significant. (**H**) DE genes contributing to the “TGFβ signaling pathway” from the DEA “ad-KLF4 vs. ad-Null” in SScL fibroblasts. Red: upregulated DE genes, blue: downregulated DE genes. (**I**) Validation of EGR1 by Western blotting in ad-KLF4 infected SScL fibroblasts. (**J**) Effect of ad-KLF4 (ad-K) and ad-Null (ad-N) on KLF4, αSMA, and collagen type I protein abundance in NL fibroblasts stimulated with TGFβ1 (TGF) or vehicle (veh) for 72 h. The data was normalized to GAP and the fold change shown is relative to ad-Null with vehicle (ad-N+veh) for each cell line. Representative Western blots are shown below each graph. A RM one-way ANOVA with uncorrected Fisher’s LSD comparisons was performed to compare ad-K+veh and ad-N+TGF to ad-N+veh as well as ad-K+TGF to ad-N+TGF. * *p* < 0.05, ** *p* < 0.01; ns: not significant.

From our DEA “ad-KLF4 vs. ad-Null” in SScL fibroblasts, we took our list of DEGs and merged it with the list of fibroblast markers extracted from Tsukui et al. [15] to make a heatmap based on our counts (Figure 5A). This heatmap reveals that in SScL fibroblasts overexpressing KLF4 (1) three markers of alveolar fibroblasts are upregulated (GPX3, PRELP, SCUBE2), (2) three markers of inflammatory fibroblasts are downregulated (CCL2, HIF1A, SERPINE1), and (3) two markers of fibrotic fibroblasts are downregulated (CTHRC1, TNC). Differential expressions of selected genes out of each group were validated by qPCR, as follows: GPX3 and SCUBE2 were upregulated, albeit not significantly for SCUBE2 due to a wide range in the dataset (*p* = 0.0745) (Figure 5B), and both CCL2 and SERPINE1 were significantly downregulated in KLF4-expressing SScL fibroblasts (Figure 5C), consistent with our RNAseq data. The decrease in SERPINE1 protein abundance was confirmed in the conditioned media of KLF4-expressing SScL fibroblasts (Figure 5C). We also confirmed the downregulation of CTHRC1 at both the mRNA and protein levels in whole cell lysates and conditioned media of ad-KLF4 SScL fibroblasts (Figure 5D). The downregulation of IL6, a well-known inflammatory cytokine that plays a role in SSc-associated fibrosis, was also validated at both the mRNA and protein levels (Figure 5E).

As functional readouts, we assessed apoptosis and cell proliferation by quantifying in ad-KLF4-infected SScL fibroblasts the cleaved form of caspase 3 (CASP3), and measuring proliferation using the 3-(4,5-dimethylthiazol-2-yl)-2,5-diphenyltetrazolium bromide (MTT) assay, respectively (Figure 5F,G). Western blot of CASP3 revealed a significant decrease in both full and cleaved forms of CASP3 in ad-KLF4 SScL fibroblasts (Figure 5F), suggesting that apoptosis is not active in these fibroblasts. The MTT assay showed a significant increase in proliferation in ad-KLF4 SScL fibroblasts (Figure 5G). Together these results highlight that KLF4-OE enhances proliferation while reducing apoptosis in SScL fibroblasts.

From the “TGF-beta signaling pathway” (Figure 5H and Figure S2), we captured the upregulation of the anti-fibrotic factors *ID1* and *CDKN2B*, along with the downregulation of the pro-fibrotic factors *GREM1*, *RGMB*, *MYC*, *THBS1*, *INHBA*, *TGFBR1*, and *THSD4*. We also observed a noticeable, albeit not significant, decrease in EGR1 protein abundance in ad-KLF4-infected SScL fibroblasts (Figure 5I). We also tested whether KLF4-OE could prevent TGFβ1-induced upregulation of αSMA and collagen type I in normal primary human fibroblasts. Efficiency of the adenoviral KLF4 expression was confirmed (Figure 5J). KLF4-OE significantly attenuated the TGFβ1-induced increase in αSMA and collagen type I (Figure 5J). Together these results show that restoring KLF4 levels prevents TGFβ1-induced fibrogenesis.

To determine if KLF4 expression follows a gradient pattern across alveolar, inflammatory, and fibrotic fibroblasts, we accessed GSE132771and GSE128169 datasets to track KLF4 within these three subtypes and by condition (normal and SSc-ILD lungs) (Figure S3). Because the alveolar subtype is lost in SSc-ILD lungs in the Tsukui dataset, while the inflammatory and fibrotic fibroblasts were over-represented (Figure S3A–C), tracking KLF4 levels by subtype and condition was not possible. However, in the Valenzi dataset, all subtypes were well represented (Figure S3D–F), making KLF4 tracking possible. Weighted KLF4 contribution was calculated as mean KLF4 expression per subtype multiplied by the proportion of that subtype within each condition (Figure S3G) and this analysis shows that (1) alveolar and inflammatory fibroblasts have the highest weighted KLF4 contribution in normal lungs while fibrotic fibroblasts have the lowest, and (2) the fibrotic subtype has the highest contribution in SSc-ILD lungs, followed by inflammatory subtype, and alveolar subtype last. Our data also show that the contribution of the alveolar and inflammatory subtypes drop noticeably in SSc-ILD as compared to NL lungs, while it increases in the fibrotic subtype. To determine how much KLF4 is present in the fibroblast population as a whole in normal and SSc-ILD lungs, we calculated the total fibroblast KLF4 load by summing KLF4 contibutions across all subtypes within each condition (Figure S3H), and captured that KLF4 is decreased in the fibroblasts of SSc-ILD lungs as compared to normal lungs, results that are consistent with the levels we captured in cultures of NL and SScL fibroblasts (Figure 2A).

**Figure 6:**
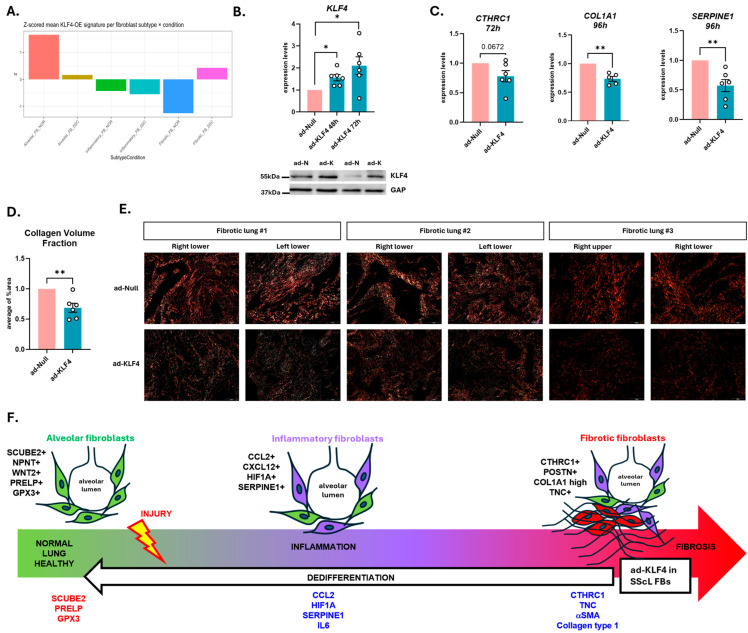
Effect of KLF4 restoration on TGFβ signaling pathway. (**A**) Comparison of the KLF4-OE signature in SScL fibroblasts to the endogenous signatures observed in alveolar, inflammatory, and fibrotic fibroblasts from normal and SSc lungs in GSE128169. Z-scored mean of the KLF4-OE signature represents degree of resemblance. (**B**–**D**) Effect of restoring KLF4 levels by adenoviral infection in fibrotic human lung tissue (organ culture model) on (**B**) the mRNA (48 h, 72 h) and protein levels of KLF4 (72 h), (**C**) CTHRC1 (72 h), COL1A1, and SERPINE1 (96 h) mRNA levels, and (**D**) collagen volume fraction levels, expressed in average of the % area (n = 8–12), based on Picro Sirius Red (PSR) stained sections imaged under polarized light. Pink: ad-Null samples, turquoise: ad-KLF4 samples. * *p* < 0.05, ** *p* < 0.01 relative to ad-Null. (**E**) Representative PSR stained sections of ad-KLF4 and ad-Null infected lung cores from 3 human fibrotic lungs. (**F**) Model of sequential differentiation of alveolar fibroblast lineage after injury adapted from Tsukui et al. [15] along with the signature of dedifferentiation identified in primary human SScL fibroblasts when KLF4 was overexpressed. Genes in red are upregulated and genes in blue are downregulated in our dataset.

We performed another analysis using the Valenzi dataset to determine if the signature we obtained by overexpressing KLF4 (KLF4-OE) in SScL fibroblasts was similar to any of the endogenous subtypes captured in normal and SSc lungs. We entered all 614 DE genes that our total RNAseq analysis returned from KLF4-OE in SScL fibroblasts, and calculated the mean module score for each subtype by condition group, and z-scored these values across groups. This analysis revealed that normal alveolar fibroblasts have the highest score, meaning that the KLF4-OE signature in SScL fibroblasts resembles normal alveolar subtype the most (Figure 6A), followed by SSc-ILD fibrotic fibroblasts, SSc-ILD alveolar fibroblasts, NL inflammatory fibroblasts, SSc-ILD inflammatory fibroblasts, and NL fibrotic fibroblasts. The results of this analysis support our conclusions that KLF4-OE transitions SScL fibroblasts towards the homeostatic phenotype of NL alveolar fibroblasts, the precursors of this lineage.

### 3.5. Organ Culture Model: KLF4-OE in Fibrotic Human Lungs

The effects of restoring KLF4 levels on fibrosis were also investigated in the human organ culture model that recapitulates the cellular complexity of whole lungs [22]. Explanted fibrotic lungs were acquired and processed to generate 4–5 mm^2^ cores that were cultured with ad-KLF4 and ad-Null for 48 h, 72 h, and 96 h. KLF4-OE was confirmed at the mRNA and protein levels in the cores (Figure 6B). Restoring KLF4 in fibrotic lung cores downregulated the mRNA levels of *CTHRC1*, *COL1A1*, and *SERPINE1* as compared to ad-Null (Figure 6C), and significantly decreased collagen volume fraction (Figure 6D,E). These results obtained from the organ culture model show that restoring KLF4 in explanted fibrotic human lung tissues reduced fibrosis by repressing pro-fibrotic genes, highlighting that KLF4 can reverse established lung fibrosis.

Together, our results highlight that overexpression of KLF4 initiates dedifferentiation and shifts the phenotype of fibrotic and inflammatory fibroblasts towards the phenotype of their lineage precursors, namely alveolar fibroblasts, by (1) downregulating fibrotic (CTHRC1, TNC, αSMA, collagen type I) and inflammatory markers (CCL2, HIF1A, SERPINE1, IL6) while upregulating markers of alveolar fibroblasts (SCUBE2, PRELP, GPX3) (Figure 6F), (2) promoting proliferation while decreasing apoptosis, and (3) repressing TGF*β* signaling. Initiating this dedifferentiation cascade in fibroblasts may contribute to the reversal of advanced fibrosis in human whole lung tissue.

## 4. Discussion

Our results show that restoring KLF4 levels in primary human SScL fibroblasts initiated dedifferentiation of αSMA expressing myofibroblasts, a process characterized by the loss of αSMA stress fibers and recognized to be required for fibrosis resolution [23,24]. Several therapeutic strategies have been shown to induce dedifferentiation of myofibroblasts including PGE2, FGF2, cAMP, and Cherenkov photodynamic therapy [23,25]. Our data suggest that KLF4 is also a dedifferentiation factor. Kato et al. [24] demonstrated that dedifferentiation is impaired by aging processes, explaining why fibrosis seems persistent and harder to reverse in older individuals. Interestingly, KLF4 expression levels have been shown to decline with aging, a phenomenon associated with immune dysfunction and deterioration of vascular health and tissue repair [26,27,28]. These reports in conjunction with our data suggest that loss of KLF4 may contribute to age-associated fibrosis persistence, and that boosting KLF4 levels could potentially promote fibrosis resolution and improve vascular and immune function in aging tissues. Definitive confirmation of this model will require future mechanistic and in vivo validation.

The enrichment and impact analysis done using iPathwayGuide revealed that markers of myofibroblasts, EMT, and inflammation decline with overexpression of KLF4 in SScL fibroblasts, and that a shift occurs towards the alveolar fibroblast cell type as described by Travaglini et al. [21]. This was confirmed with the comparison we made between the transcriptomic signature of ad-KLF4 infected SScL fibroblasts and the markers of alveolar, inflammatory, and fibrotic fibroblasts defined by Tsukui et al. [15].

The markers of fibrotic fibroblasts, CTHRC1 and TNC, were both downregulated in ad-KLF4-infected SScL fibroblasts. This is meaningful since CTHRC1 is now considered the best marker of ECM-high-producing fibroblasts based on studies conducted in lung fibrosis, myocardial infarction, and COVID-19 disease [14,29,30], and TNC is a well-known marker of chronic fibrotic fibroblasts that promotes fibroblast proliferation, migration, and differentiation into myofibroblasts that is consistently upregulated in fibrotic lung diseases [31,32,33]. To capture the downregulation of both CTHRC1 and TNC upon KLF4-OE in primary human SScL fibroblasts is remarkable and suggests that the fibrotic fibroblasts are undergoing dedifferentiation.

The same might be true for the inflammatory fibroblasts, as we captured downregulation of CCL2, HIF1A, and SERPINE1 in our dataset. CCL2 recruits monocytes and macrophages, and by doing so it sustains leukocyte infiltration [31,34], while HIF1A is a hypoxia-induced transcription factor that drives stress-induced inflammatory signaling and fibroblast-to-myofibroblast differentiation that contributes to lung fibrosis [35,36]. SERPINE1 is known for promoting ECM remodeling and inflammation by reducing fibrinolysis and enhancing cytokines in lung fibrosis and SSc [37,38,39]. It also contributes to pulmonary aging [37]. By overexpressing KLF4 in SScL fibroblasts, a significant decrease in all three markers was observed, suggesting a reversal of the inflammatory fibroblast phenotype. This is consistent with silencing of KLF4 in murine airway smooth muscle cells leading to upregulation of CCL2 expression, confirming a negative correlation between KLF4 and CCL2 [40].

Our data highlight that the fibrotic and inflammatory fibroblasts may be regressing back to the phenotype of their lineage precursors, namely the alveolar fibroblasts [15]. Indeed, the combined upregulation of SCUBE2, PRELP, and GPX3 upon expression of KLF4 in SScL fibroblasts seems to validate this theory. SCUBE2 is a reliable marker of lung-specific alveolar fibroblast lineage that gives rise to the inflammatory and fibrotic fibroblasts upon injury [15]. Prior to injury, alveolar fibroblasts provide trophic support to alveolar epithelial cells such as AT2 cells [41]. PRELP is another good marker, as it is necessary to maintain proper alveolar matrix and stabilize epithelial niche [42]. The antioxidant GPX3 is another marker of alveolar fibroblasts that protects the epithelial cells from oxidative stress and supports the redox balance in the alveolar niche [43,44]. The fact that all three of these markers were upregulated in our dataset confirms that full dedifferentiation from fibrotic/inflammatory fibroblasts to alveolar fibroblasts is initiated in ad-KLF4 infected SScL fibroblasts.

We also uncovered a new anti-inflammatory effect of KLF4 in SScL fibroblasts, as we observed a significant decrease in IL6 levels. KLF4 has a double-faceted role in inflammatory diseases, able to both enhance and repress inflammation depending on the environment [45]. For example, when bound to the IL10 promoter, KLF4 leads to an anti-inflammatory response in human lung epithelial cells [46], but in polymorphonuclear neutrophils (PMNs), KLF4 amplifies the pro-inflammatory cytokines TNFα and IL8 while simultaneously decreasing IL10 [47]. In dendritic cells, KLF4 modulates the inflammatory response and IL6 expression due to its dual role as a transcription factor and a chromatin remodeler [48], as follows: (1) as a transcription factor, KLF4 binds to specific proximal CACCC sites on IL6 promoter, and (2) as a chromatin remodeler, by increasing the acetylation status of histone H4 in the proximal region of the IL6 promoter. The inflammation associated with rheumatoid arthritis seems to result from KLF4 transcriptional activation of fibroblast-like synoviocytes by direct interaction with nuclear factor-kappa B (NFκB) [49]. KLF4 can also be acetylated by lysine acetyltransferase 2B (KAT2B aka PCAF), a modification that enhances its ability to regulate IL23, IL36a, and IL6 [50,51]. Here we show that in primary human SScL fibroblasts, restoring KLF4 levels significantly lowers IL6 levels, reinforcing the ability of KLF4 to reduce inflammation.

KLF4 restoration in SScL fibroblasts enhanced cell proliferation while decreasing apoptosis, suggesting that KLF4 exerts its anti-fibrotic effects not by globally suppressing fibroblast survival, but by reprogramming SSc fibroblasts towards a less fibrogenic state despite preserved or even enhanced viability. This is consistent with the emerging view that KLF4 functions as a context-dependent brake on fibroblast activation rather than a simple pro-apoptotic factor [5,7].

We also examined the effects of restoring KLF4 levels in a fibrotic human lung organ culture model. This model retains the full complexity of the cellular make-up of the tissue of interest, including the ECM stiffness and collagen crosslinking [22]. By using human lung tissue cores, we ensured that our observations are directly relevant to human disease biology. The reduction in collagen volume fraction following KLF4 overexpression in end-stage human pulmonary fibrosis explants suggests that restoring KLF4 may attenuate ECM accumulation even in advanced fibrosis. The proposed antifibrotic effect of KLF4 on established fibrosis warrants further experimental validation in both human ex vivo and murine in vivo models.

## 5. Conclusions

This study is the first to report the impact of KLF4-OE in primary human SScL fibroblasts and provide insights into the functional enrichment and impacted pathways downstream of KLF4. Our results suggest that KLF4 initiated dedifferentiation of SScL fibroblasts, shifting their identity towards the alveolar fibroblast phenotype. Our results not only confirm the anti-fibrotic role of KLF4, they also emphasize its anti-inflammatory properties and suggest that restoration of KLF4 can reverse established fibrosis in human lung tissues, warranting further validation and exploration of targeted therapeutic strategies aiming at restoring KLF4 levels in fibroblasts.

## Figures and Tables

**Table 1:** Information on SSc patients and normal (NL) donors.

Code	Age (yrs)	Ancestry	Gender	FVC	PA Mean	Allocation
SSc-18	42	African	Female	46	24	NL/SScL baseline
SSc-23	52	European	Female	36	22	NL/SScL baseline
SSc-25	52	European	Female	23	20	NL/SScL baseline
SSc-38	49	European	Female	26	21	NL/SScL baseline
SSc-113	67	European	Female	60	24	NL/SScL baseline
SSc-27	42	European	Female	42	15	NL/SScL baseline; ad-KLF4/RNAseq
SSc-30	59	European	Female	39	19	NL/SScL baseline; ad-KLF4/RNAseq
SSc-53	46	European	Male	52	22	ad-KLF4/RNAseq
SSC-66	51	European	Male	70	25	ad-KLF4/RNAseq
SSc-87	64	European	Male	56	23	ad-KLF4/RNAseq
NL-45	42	European	Female	n/a	n/a	NL/SScL baseline
NL-57	60	European	Female	n/a	n/a	NL/SScL baseline
NL-78	63	African	Female	n/a	n/a	NL/SScL baseline
NL-139	32	European	Female	n/a	n/a	NL/SScL baseline
NL-59	50	European	Female	n/a	n/a	NL+TGF
NL-122	19	Unknown	Female	n/a	n/a	NL+TGF
NL-129	51	European	Female	n/a	n/a	NL+TGF
NL-143	29	African	Female	n/a	n/a	NL+TGF
NL-38	37	European	Female	n/a	n/a	NL/SScL baseline; NL+TGF
NL-66	63	European	Female	n/a	n/a	NL/SScL baseline; NL+TGF
NL-41	53	European	Female	n/a	n/a	NL/SScL baseline; NL+TGF; NL+TGF/ad-KLF4
NL-94	52	European	Male	n/a	n/a	NL+TGF; NL+TGF/ad-KLF4
NL-81	33	European	Female	n/a	n/a	NL+TGF/ad-KLF4
NL-127	46	African	Male	n/a	n/a	NL+TGF/ad-KLF4

FVC: forced vital capacity, PA mean: arterial pressure mean, n/a: not available, NL/SScL baseline shown in Figure 2A,B, NL+TGF: NL fibroblasts treated with TGFβ shown in Figure 2C, ad-KLF4/RNAseq: SScL fibroblasts infected with ad-KLF4 and sent to RNAseq shown in Figures 3 and 5, NL+TGF/ad-KLF4: NL fibroblasts infected with ad-KLF4 and treated with TGFβ shown in Figure 5J.

## Data Availability

Data are available from the corresponding authors upon reasonable request. The RNAseq data generated in this study have been deposited in the NCBI Gene Expression Omnibus (GEO) under accession number GSE317058.

## Supplementary Materials

The following supporting information can be downloaded at: https://www.mdpi.com/article/10.3390/cells15100921/s1, Figure S1: DEseq2 output figures; Figure S2: TGF-BETA signaling pathway; Figure S3: KLF4 in fibroblasts subtypes in 2 single-cell RNAseq datasets; Table S1: DE genes in ad-KLF4 SScL fibroblasts; Table S2: Impacted pathways in ad-KLF4 SScL fibroblasts.

## Abbreviations

The following abbreviations are used in this manuscript:

SSc	Systemic sclerosis
SSc-ILD	SSc-associated interstitial lung disease
EMT	epithelial-mesenchymal transition
AECs	alveolar epithelial cells
SScL	human primary SSc lung
NL	normal lung
ad-KLF4	KLF4 adenovirus
ad-Null	null adenovirus
DEA	differential expression analysis
DEGs	differentially expressed genes
KLF4-OE	KLF4 overexpression
RNAseq	RNA sequencing
MTT	3-(4,5-dimethylthiazol-2-yl)-2,5-diphenyltetrazolium bromide
