# Supplementary material for: KLF4 Initiates Dedifferentiation of Systemic Sclerosis Lung Fibroblasts
Source: Cells. 2026 May 18;15(10):921. doi: 10.3390/cells15100921 (PMC13204799; doi:10.3390/cells15100921)
Supplement: Supplementary file 1 [file cells-15-00921-s001.zip › Supplemental Figures S1 S2 S3.pptx]

## Slide 1
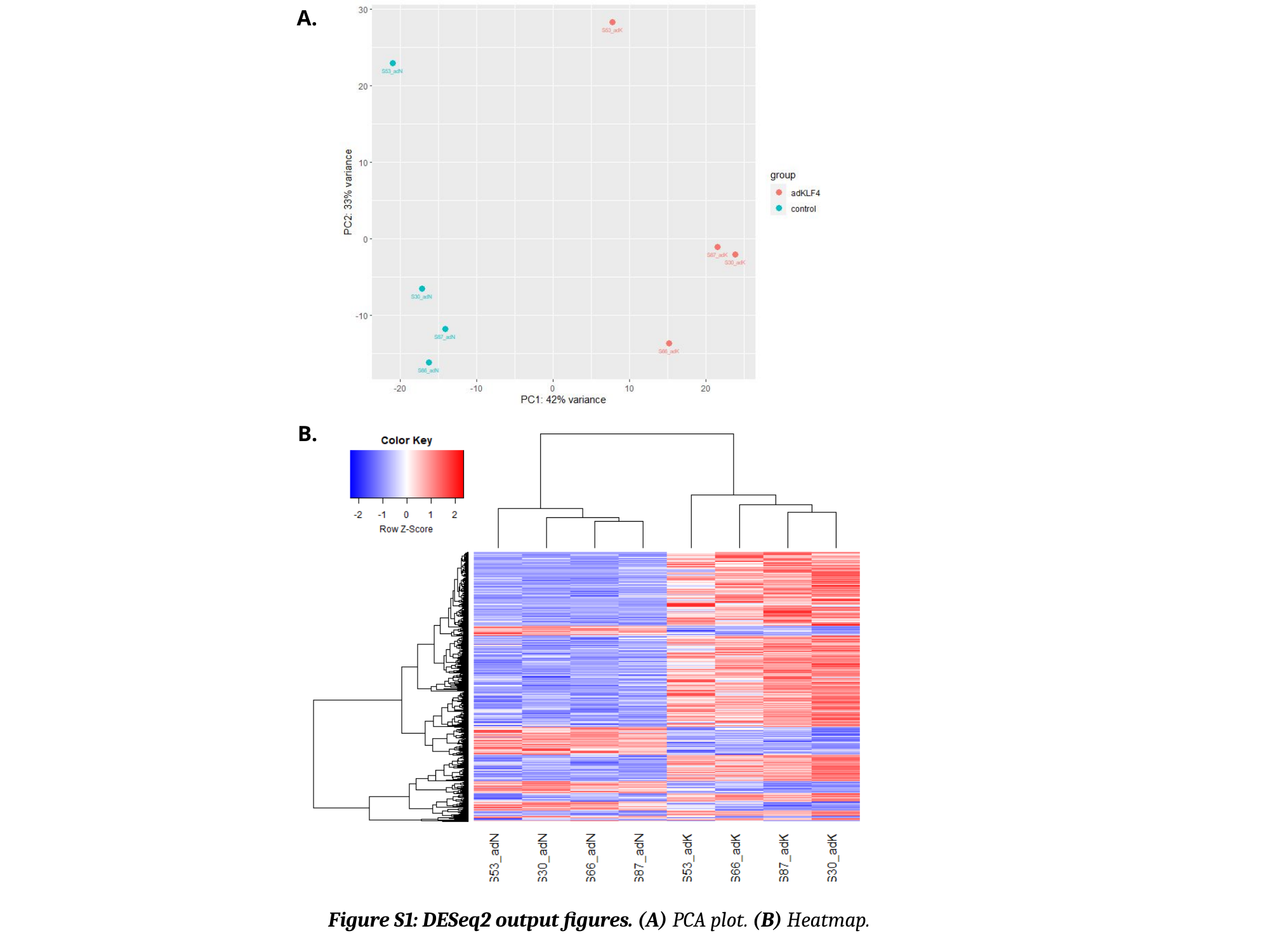

A.
B.
Figure S1: DESeq2 output figures. (A) PCA plot. (B) Heatmap.

## Slide 2
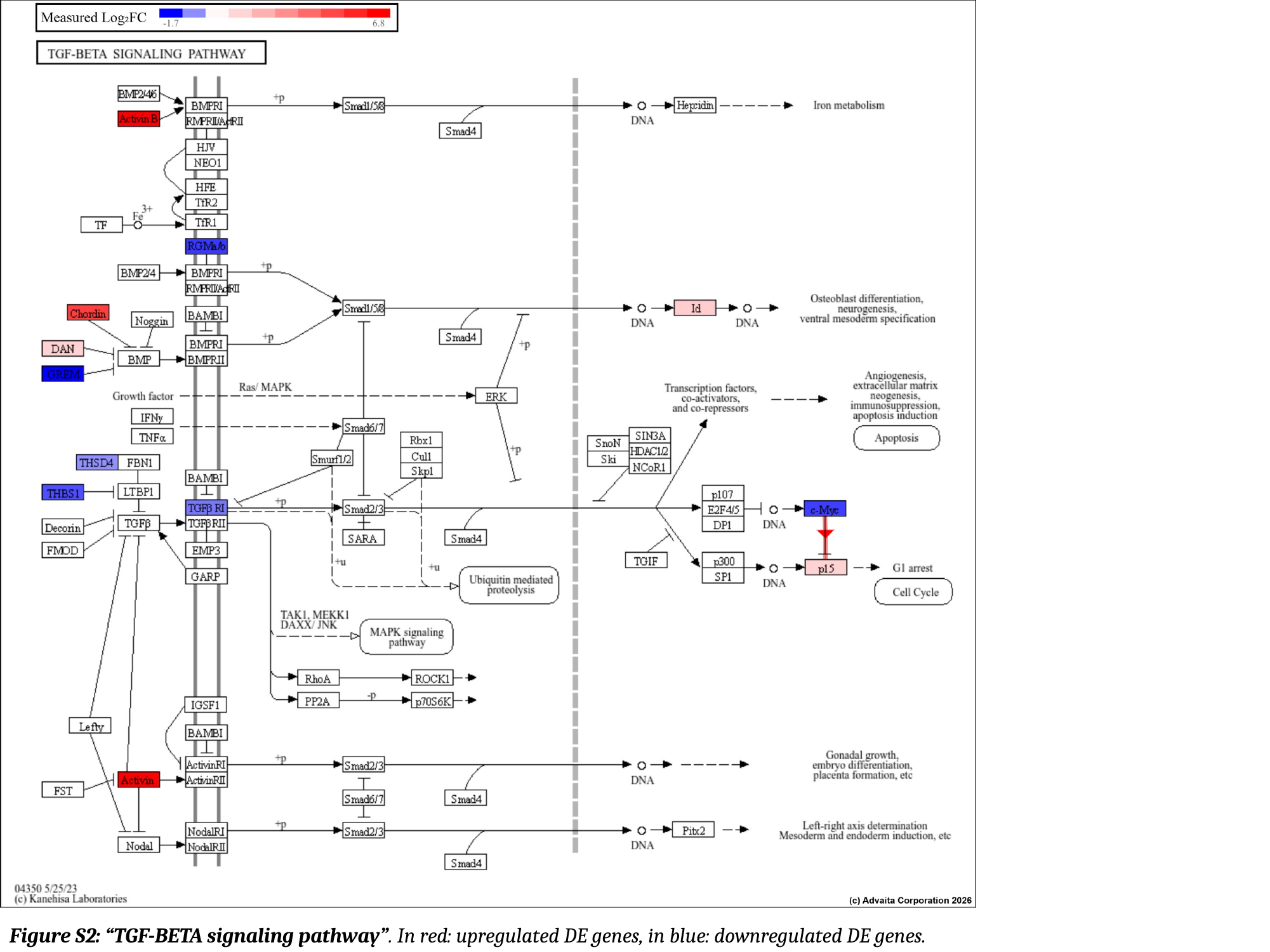

Figure S2: “TGF-BETA signaling pathway”. In red: upregulated DE genes, in blue: downregulated DE genes.

## Slide 3
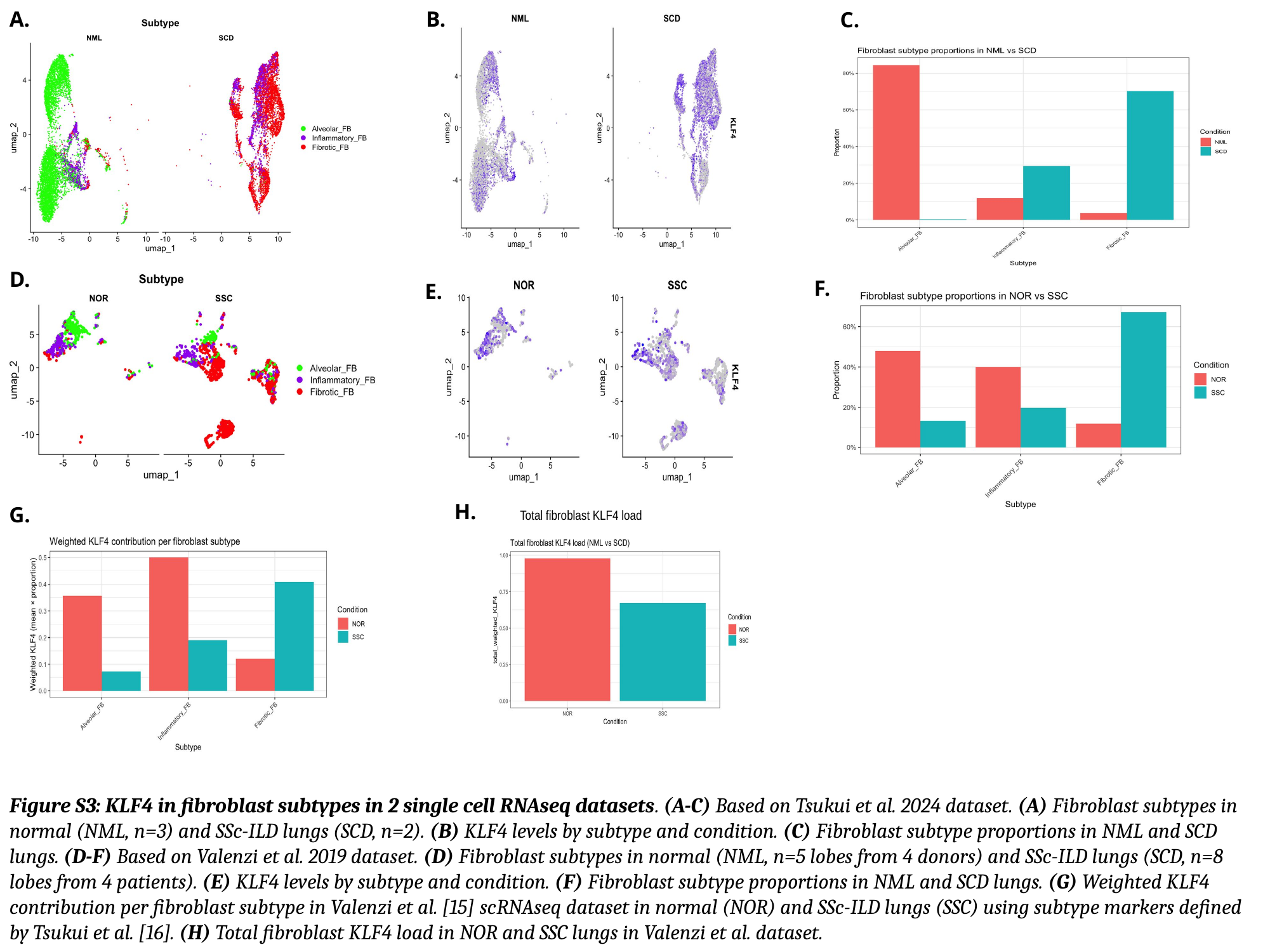

A.
B.
C.
D.
F.
E.
H.
G.
Total fibroblast KLF4 load
Figure S3: KLF4 in fibroblast subtypes in 2 single cell RNAseq datasets. (A-C) Based on Tsukui et al. 2024 dataset. (A) Fibroblast subtypes in normal (NML, n=3) and SSc-ILD lungs (SCD, n=2). (B) KLF4 levels by subtype and condition. (C) Fibroblast subtype proportions in NML and SCD lungs. (D-F) Based on Valenzi et al. 2019 dataset. (D) Fibroblast subtypes in normal (NML, n=5 lobes from 4 donors) and SSc-ILD lungs (SCD, n=8 lobes from 4 patients). (E) KLF4 levels by subtype and condition. (F) Fibroblast subtype proportions in NML and SCD lungs. (G) Weighted KLF4 contribution per fibroblast subtype in Valenzi et al. [15] scRNAseq dataset in normal (NOR) and SSc-ILD lungs (SSC) using subtype markers defined by Tsukui et al. [16]. (H) Total fibroblast KLF4 load in NOR and SSC lungs in Valenzi et al. dataset.
